# Validation and Cultural Adaptation of Explanatory Model Interview Catalogue (EMIC) in Assessing Stigma among Recovered Patients with COVID-19 in Saudi Arabia

**DOI:** 10.3390/ijerph18168261

**Published:** 2021-08-04

**Authors:** Lamia A. Al-Zamel, Shatha F. Al-Thunayan, Afnan A. Al-Rasheed, Munirah A. Alkathiri, Faisal Alamri, Faleh Alqahtani, Amer S. Alali, Omar A. Almohammed, Yousif A. Asiri, Adel S. Bashatah, Yazed AlRuthia

**Affiliations:** 1 King Faisal Specialist Hospital and Research Centre, Department of Pharmaceutical Care, Riyadh 11564, Saudi Arabia; lalzamel@kfshrc.edu.sa; 2 Department of Clinical Pharmacy, College of Pharmacy, King Saud University, Riyadh 11451, Saudi Arabia; shathaalthunayan@gmail.com (S.F.A.-T.); 436200445@student.ksu.edu.sa (M.A.A.); yasiri@KSU.EDU.SA (Y.A.A.); 3 Department of Pharmaceutical Care, Prince Sultan Military Medical City, Riyadh 12233, Saudi Arabia; Afnanalrasheed@gmail.com; 4 Basic Sciences Department, College of Science and Health Professions, King Saud bin Abdulaziz University for Health Sciences, Jeddah 22384, Saudi Arabia; alamrif@ksau-hs.edu.sa; 5 King Abdullah International Medical Research Center, Jeddah 22384, Saudi Arabia; 6 Department of Pharmacology and Toxicology, College of Pharmacy, King Saud University, Riyadh 11451, Saudi Arabia; afaleh@ksu.edu.sa; 7 Department of Pharmaceutics, College of Pharmacy, Prince Sattam bin Abdulaziz University, Al-Kharj 16278, Saudi Arabia; a.alali@psau.edu.sa; 8 Pharmacoeconomics Research Unit, College of Pharmacy, King Saud University, Riyadh 11451, Saudi Arabia; 9 Department of Nursing Administration & Education, College of Nursing, King Saud University, Riyadh 11451, Saudi Arabia; abashatah@KSU.EDU.SA

**Keywords:** COVID-19, social stigma, mental health, surveys and questionnaires, Saudi Arabia

## Abstract

Stigma is a negative feeling affecting many patients with various health conditions, especially the contagious ones such as COVID-19. The Explanatory Model Interview Catalogue (EMIC) is one of the valid and reliable stigma-measuring tools; however, it has not been translated and validated in Arabic. Therefore, the aim of this study was to translate and validate the EMIC in Arabic among a sample of Arabic-speaking adults who recently recovered from COVID-19 in Saudi Arabia. The 12 items of the EMIC scale were forward- and backward-translated and reviewed by all authors to check the face and content validity prior to approving the final version of the Arabic 12-item EMIC. A total of 174 participants aged ≥18 years who contracted COVID-19 and recovered as of 29 July 2020 were interviewed. The Cronbach’s alpha of the Arabic version of the 12-item EMIC was 0.79, indicating an acceptable level of internal consistency. Using principal component analysis with varimax rotation, two factors explained more than 60% of the variance of the translated EMIC scale. The mean EMIC score was 5.91, implying a low level of stigma among participants. Married participants (*β* = 2.93; 95%CI 0.88 to 4.98, *p* = 0.005) and those with a family history of mental illness (*β* = 2.38; 95%CI 0.29 to 4.46, *p* = 0.025) were more likely to have higher EMIC scores in comparison to their counterparts who were unmarried and had no family history of mental illness. On the contrary, older adults were less likely to have high EMIC scores (*β* = −0.11; 95%CI −0.21 to −0.01, *p* = 0.03). Future studies with larger samples of patients with COVID-19 and various health conditions should be conducted to examine the validity and reliability of the Arabic version of the EMIC among different patient populations and to unveil the factors that may play a role in patients’ feelings of stigmatization in this part of the world.

## 1. Introduction

The coronavirus disease (COVID-19) is caused by a novel coronavirus known as severe acute respiratory syndrome coronavirus 2 (SARS-CoV-2), where the first cases were identified in Wuhan, China in 2019, and, since then, it has spread rapidly throughout the world, causing universal panic [1]. It had and is still having a large damaging effect on healthcare systems around the world, affecting mental, physical, and social health, as well as having a detrimental impact on the global economy [2]. In order to contain this pandemic and the transmission of the infection, physical distancing and quarantine were necessary to break the chain of infection, learning from the examples of other previous infections, such as plague, yellow fever, tuberculosis (TB), and Ebola virus. However, these precautionary measures may have resulted in stigma and discrimination toward individuals infected with SARS-CoV-2 [3,4].

The fear of this new virus has brought a whole range of negative perceptions and behaviors, such as stigma, as people who have been infected, or were in close contact with patients infected with SARS-CoV-2, were stigmatized by people around them [5]. In March 2020, the World Health Organization (WHO) reported that communities feared and stigmatized not only patients but also healthcare workers, as many believe they are a possible source of infection [6]. Several incidents of racism and stigmatization against COVID-19 survivors, family members, and healthcare workers have been widely reported, particularly among Chinese and Italian people, who have faced hate attacks [7].

Stigma is a complex construct that refers mainly to the negative social perceptions toward certain individuals and their families based on the identification of a disvalued health or social problem. Generally, it can be categorized into two types: self-stigma and perceived stigma. Self-stigma means one’s own beliefs and negative attitudes to one’s self-image because of society’s views toward his illness, while perceived stigma refers to the negative attitude and perception that the society maintains toward people with certain illnesses or social issues [8]. During the SARS-CoV-2 outbreak, the incidence rates of various mental illnesses, such as depression and anxiety, increased, especially among elderly and healthcare workers [1,9]. These mental illnesses may have been triggered by certain factors such as stigma, self-isolation, fear of infection, financial loss, and the uncertainty about their health status [5,9,10]. A negative perception toward SARS-CoV-2 infection can result in shame, leading infected individuals to hide their symptoms and avoid or delay seeking medical attention, which may worsen their mental and physical health, as well as the mental health of their families, friends, or caregivers [5].

Several instruments have been developed to understand the barriers and social burden of infection-related stigma [11]. One of the well-studied instruments is the Explanatory Model Interview Catalogue (EMIC), which was developed by Weiss et al. as a tool to understand the perceptions, beliefs, and practices related to leprosy [12]. It was built as a framework for social analysis that consists of four key themes: patterns of distress that are related to patients’ concerns regarding their illness (e.g., treatment outcome, social and economic implications, and most troubling aspect of their illness), perceived causes (e.g., the patient perceives the cause of illness based on their religious, socio-economic, and cultural background), help-seeking patterns (e.g., patient history of treatment and support-seeking from various professional and nonprofessional providers), and general illness beliefs and behaviors (e.g., societal and cultural beliefs about illness and their impact on individuals’ behaviors). The original scale is composed of twelve simple questions that were found helpful in assessing stigma and easing the data collection process [12,13]. The validity and reliability of EMIC as a stigma assessment tool have been tested and confirmed in different settings [14,15,16]. The social stigma in the Indonesian population with leprosy was assessed by Peters et al. using the EMIC [17]. The cross-culturally adapted EMIC was found to be valid in assessing various aspects of social stigma among leprosy survivors in Indonesia. Furthermore, Peters et al. [17] recommended the adaptation of EMIC to other neglected tropical diseases. In Nigeria, the EMIC was used to explore the predictors of TB-associated stigma among a sample of urban and rural patients in the state of Lagos. The EMIC scale demonstrated good validity and reliability in assessing Tb-associated stigma among a sample of 790 Nigerians. Moreover, unmarried individuals had higher odds of having a higher stigma score in comparison to their married counterparts. In addition, those aged ≥35 years and with higher incomes had higher odds of having high stigma scores [18].

Understanding the infectious disease related stigma could help in effectively managing the psychological impact of infectious diseases and containing their transmission. Therefore, the local adaptation of the EMIC scale in the current COVID-19 pandemic can provide an insight into the impact of this pandemic on those affected by the disease and eventually help policymakers address the unintended effects of different precautionary measures taken to contain the spread of infection. In addition, such information should help guide the clinical practice in assessing the need for psychological support during such a pandemic. Therefore, the aim of this study was to validate and culturally adapt an Arabic-translated version of the 12-item EMIC among a sample of unhospitalized COVID-19 survivors in Saudi Arabia.

## 2. Materials and Methods

### 2.1. Study Design and Setting

This was a telephone interview-based cross-sectional study that took place between the 19th of July and the 30th of December 2020. Adult patients aged 18 years and above who understand Arabic and had recently recovered from COVID-19 that was confirmed by a polymerase chain reaction (PCR) test for SARS-CoV-2 during their infection were identified using the HESN database, which is the national database for documenting all COVID-19 infections in Saudi Arabia. Patients with asymptomatic SARS-CoV-2 infection were excluded from the study. Those who met the inclusion criteria were randomly selected from the overall population of COVID-19 survivors and were contacted over the phone and informed about the purpose of the study, and, if they agreed to participate in the study, their verbal consent was obtained. Upon the reception of their consent to participate in the study, participants were interviewed over the phone by four trained healthcare providers for 10 to 15 min.

### 2.2. Instrument and Measurement

The permission to translate and validate the English version of the EMIC into Arabic was obtained from Weiss et al. via email [12]. The EMIC consists of 12 questions covering different feelings and experiences suggesting stigma and are scored on a 4-point Likert scale (“Yes” = 3; “Possibly” = 2; “Uncertain” = 1; and “No” = 0). Those who answered “Yes” are believed to have a strong and positive indication of stigma, and thus, their response has the highest value (e.g., three points); meanwhile, those with a “No” response is believed not to have any stigma feelings and, hence, their response is assigned with the lowest value (e.g., zero point). However, question number two has a reverse score. The EMIC total score ranges from 0 to 36 with higher scores indicating higher levels of stigma. The English and Arabic-translated version of the EMIC is shown in Appendix A.

### 2.3. EMIC Translation and Validation

Forward translation was conducted by two bilingual healthcare providers and reviewed by a certified English translator whose native language is Arabic, and the backward translation was conducted by a bilingual healthcare provider whose native language is English. The final Arabic version of the translated EMIC was reviewed by all coauthors to check the face and content validity, and they approved it after no significant difference was found between the forward- and backward-translated versions of the EMIC and the original scale.

### 2.4. Data Collection and Ethical Approval

Besides the 12 items from the translated EMIC scale that were used to assess stigma among recovered COVID-19 patients, sociodemographic characteristics (e.g., gender, age, marital status, number of family members living with the recovered patient, employment status, monthly income, region of residence, and educational level) and medical characteristics (e.g., presence of comorbidities, such as diabetes and hypertension, and family history of mental illness) were collected. Furthermore, participants’ health literacy was checked using the Arabic version of the single-item literacy screener (SILS) where they were asked about their need for help to understand a prescription medication leaflet with five possible answers (5—always, 4—often, 3—sometimes, 2—rarely, 1—never). Those who answered “always,” “often,” or “sometimes” are believed to have limited/marginal health literacy, and those who answered “rarely” or “never” are believed to have adequate health literacy [19].

The ethical principles of the Helsinki declaration were adhered to throughout the data collection, storage, and analysis process, and no personal identifiers (e.g., name and address) were collected [20]. The study was approved by the central research ethics committee at the Saudi Ministry of Health, Riyadh, Saudi Arabia (IRB No: 20-11E/17-06-2020). The need for written consent was waived by the ethical committee.

### 2.5. Statistical Analysis

The internal consistency of the translated EMIC was checked using the Cronbach’s alpha method [21]. Construct validity of the Arabic-translated EMIC was examined using principal component analysis with varimax rotation. Although EMIC is used as a one-dimensional scale, there are studies that have confirmed the potential of a two-dimensional model of the EMIC [22]. Therefore, factors with an eigenvalue of >1 were extracted [23]. Additionally, confirmatory factor analysis (CFA) was conducted to examine whether or not more than one scale or factor can be extracted and used in assessing stigma among participants. The authors adhered to the good practice guidelines for translation, validation, and adaptation of questionnaires across cultures [24].

Descriptive statistics using means, standard deviations, frequencies, and percentages were performed as appropriate. Multiple linear regression to explore the relationship between EMIC stigma score and different sociodemographic (e.g., age, gender, marital status, monthly income, employment status, region of residence, educational level, and health literacy) and medical characteristics (e.g., presence of comorbidities and family history of mental illness) was conducted [22,25,26,27]. The minimum sample size was estimated to be 104 participants for multiple linear regression with up to nine independent variables and a medium effect size (Cohen’s f^2^ = 0.15) assuming a response rate of 50%. All analyses were conducted using SAS^®^ version 9.4 (SAS institute, Cary, NC, USA).

## 3. Results

Out of 211 patients who were identified from the HESN database and met the inclusion criteria, 174 (82.46%) consented to participate and were interviewed. More than half of the participants (54.60%) were females, aged ≤ 35 years (68.39%), married (52.87%), employed (79.77%), living with four or more family members (62.64%), Saudi (87.36%), had an adequate level of health literacy (84.48%) with a bachelor of science degree (54.02%), and lived in the Riyadh region (52.87%), as shown in Table 1. Moreover, most participants did not have any chronic health conditions (e.g., diabetes, hypertension, and dyslipidemia) (82.18%) or family history of mental illness (82.76%).

The Cronbach’s alpha of the Arabic version of the EMIC was 0.79, indicating an acceptable level of internal consistency. The total mean score of EMIC was 5.91, indicating a low level of stigma among the surveyed participants. Individual EMIC items’ mean scores are shown in Table 2.

**Figure 1:**
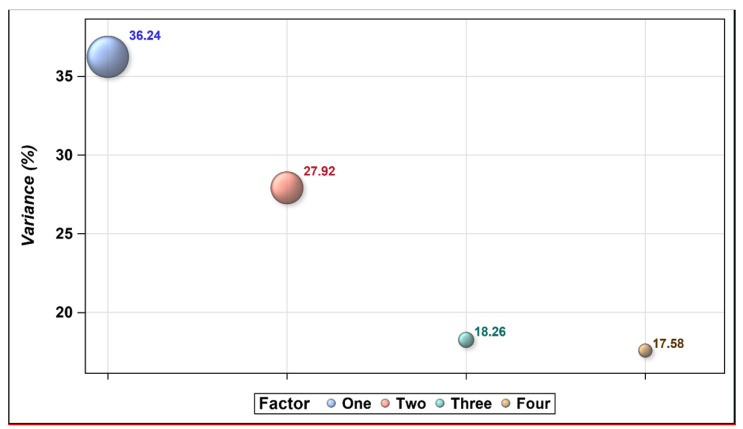
The percentage of the explained variance for each extracted factor with an eigenvalue of ≥1.

Four factors were extracted from the Arabic version of the EMIC, and the loading of each item is shown in Table 3. The Cronbach’s alphas for the extracted factors were 0.86, 0.56, 0.45, and 0.31 for factors one, two, three, and four, respectively. Moreover, factors one and two explained more than 64% of the variance in the Arabic EMIC scale, as shown in Figure 1. The CFA has shown that only one factor can be extracted from the Arabic-translated EMIC scale as the *P*-value for the chi-square test was 0.0008 lower than 0.05, indicating that the four-factor model does not fit the data. Moreover, only the items of factor-1 have shown to have significant parameter estimates with a t-value greater than 2.56.

Married participants (*β* = 2.93, *p* = 0.005) and those with a family history of mental illness (*β* = 2.38, *p* = 0.025) were more likely to have higher EMIC scores controlling for age, gender, health literacy, educational level, monthly income, employment status, and presence of comorbidities. Conversely, older adults were less likely to have high EMIC scores (*β* = −0.11, *p* = 0.03) controlling for gender, health literacy, educational level, monthly income, employment status, presence of comorbidities, family history of mental illness, and marital status, as shown in Table 4.

## 4. Discussion

Fear from the SARS-CoV-2 has brought a range of stigma and rejection in some countries around the world. People who have been infected or were in close contact with COVID-19 patients were stigmatized by some members of their own communities [5]. Healthcare workers who became infected or cared for infected people also suffered from social stigma in many countries [6]. However, the lack of culturally adapted scales to measure stigma in many cultures, especially among those in developing nations, such as in the Middle East, makes the management of this pandemic from different aspects (e.g., social, mental, and physical) more complicated. Although stigma scales, such as the 12-item EMIC, has been validated in different cultures, especially among leprosy patients, this scale has not been validated in Arabic [12,13]. The EMIC has also been validated to assess stigma toward healthcare providers and those with mental illnesses, such as depression and schizophrenia [14,15,16]. In addition, the EMIC scale is available in various languages but Arabic [22,25,26,27,28,29,30]. However, this scale, as well as other infectious-disease stigma scales, do not exist in Arabic, which makes it difficult to explore the stigma associated with infectious diseases, such as COVID-19. The validation and cultural adaptation of the newly created Arabic version of the EMIC scale in this study to explore the COVID-19-related stigma among individuals who caught the infection and were home-quarantined will enable health policymakers and researchers to assess the social impact of different precautionary measures taken to contain the spread of the pandemic in Arabic-speaking countries.

In this study, the 12-item EMIC was translated to Arabic and validated, and its internal reliability was assessed. Furthermore, the association between the EMIC score and different sociodemographic factors was explored. The Arabic-translated EMIC showed a very good level of internal consistency with a Cronbach’s alpha of 0.79, which is similar to the levels reported by Morgado et al. in the revised (12-item) Portuguese version of the scale (0.78) [22]. Although two factors explained more than 60% of the responses’ variance, which is consistent with previously published studies [22,30], the EMIC was used as a one-dimensional scale as recommended by Weiss et al. [12,13], and it has also been found to be appropriate in multiple studies [17,22,30]. Moreover, Morgado et al. tested the validity and internal consistency of the revised 15-item EMIC scale with two hypothesized factorial models, the one- and two-dimensional models, which are different than the one tested in our study [22]. Furthermore, the CFA that was conducted in this study confirmed that only one extracted factor with acceptable fit statistics is possible.

The mean total EMIC score found in this study was very low (mean score = 5.9) in comparison to other studies that reported far higher mean EMIC scores [14,18,29]. This might be explained by the fact that COVID-19 is mainly transmitted through exposure to respiratory fluids of individuals that have been infected in comparison to other infectious diseases, such as HIV, where its transmission is associated with certain frowned-upon behaviors by the society [31]. Furthermore, multiple local and international public awareness campaigns about the COVID-19 pandemic have been carried out to educate the public about its modes of transmission and the protective measures, such as mask wearing and social distancing, which should be adopted to lessen the risk of transmission. However, other infectious and mental illnesses, such as leprosy and depression, did not have the same breadth and quality of public educational campaigns as with COVID-19, resulting in higher levels of stigmatization [32,33]. Moreover, this is the first time to explore stigma among Arabic-speaking individuals in Saudi Arabia who survived COVID-19. Therefore, we cannot compare the EMIC stigma scores found in this study with other studies that examined stigma among other patient populations in Saudi Arabia. In addition, participants with a family history of mental illness and married participants had significantly higher EMIC scores (*β* = 2.38, *p* = 0.025 and *β* = 2.93, *p* = 0.005, respectively). The higher stigma scores among participants with a family history of mental illness could be attributed to the fact that family members of patients with mental illness are more likely to experience social stigma from the public [34,35]. Interestingly, married participants were more likely to feel stigmatized in comparison to their unmarried counterparts. This does not concord with the previously published findings from Nigeria that found married individuals were less likely to have high EMIC scores in comparison to their unmarried counterparts [18]. This could be explained by the fact that married individuals tend to be more cautious to transmit the disease to their loved ones and fear the perceptions of their household toward their illness in comparison to their unmarried counterparts who can easily isolate themselves and avoid the negative impressions that can be relayed to them by the public.

Conversely, older participants were less likely to have high EMIC scores (*β* = −0.11, *p* = 0.03). This is contrary to the findings among patients with leprosy in which older adults were more likely to feel stigmatized in comparison to their younger counterparts [22]. However, this can be explained by the respect and love that the elderly are supposed to be dealt with in our community as part of our Islamic culture [36]. Additionally, comparing the levels of stigma among patients with COVID-19 to other patient populations with infectious or mental illnesses is difficult due to the nature of these illnesses. For example, leprosy is an old bacterial disease that results in a multitude of physical deformities and disabilities if left untreated, leading to public misperceptions and negative reactions toward patients with leprosy in comparison to COVID-19 that can result in asymptomatic illness that is not identifiable by the public [37]. Furthermore, the differences in cultural perceptions and attitudes toward different illnesses, especially in Islamic societies, are not well-studied [38]. Although Islam encourages individuals to seek treatment and forbids the mistreatment and negative attitudes toward patients, especially those with infectious illnesses, social stigmatization and segregation toward individuals with certain infectious illnesses, such as HIV and leprosy, are still practiced in many Islamic societies [39]. On the other hand, employment status, income, education level, and health literacy did not have any significant effect on the stigma score.

To the best of our knowledge, this is the first study to translate and validate the EMIC scale into Arabic; therefore, it is the first to assess the level of stigma using the EMIC among an Arabic-speaking community during this unprecedented time. Although a low level of stigma was observed in this study, this research has some important implications that should be considered in the era of COVID-19. As married participants and those with a family history of mental illnesses were more likely to feel the stigma when having SARS-CoV-2 infection, their family members and community should be informed and educated to minimize the risk of stigma among those individuals. Moreover, psychiatric screening should be regularly performed among COVID-19 patients, and family history of mental illness should be taken into consideration in any health or social intervention designed to minimize the risk of transmission and expedite the recovery of those affected by COVID-19.

The findings of this study have to be seen in light of some limitations. First, the study was conducted among unhospitalized individuals who had already recovered from COVID-19. Thus, the findings shall not be generalized to hospitalized individuals or those who passed away from COVID-19. Nevertheless, exploring stigma among hospitalized patients within a short time of their recovery is difficult to perform given the difficulty in obtaining their consent to participate in the study in a culture that views research as an invasion of privacy. Secondly, the study sample was small and included a limited number of participants who were randomly selected from a national COVID-19 database of patients from different regions in Saudi Arabia. However, most of them were from the central region, which concords with the distributions of COVID-19 cases during that time period. Thirdly, performance bias cannot be ruled out despite the fact that all interviewers were trained and adhered to a standardized protocol for data collection. Finally, the survey was conducted several weeks after the patients recovered from the COVID-19 infection. Therefore, recall bias is possible. However, contacting those individuals and collecting data from them while sick was deemed culturally inappropriate by the research team; therefore, we decided to collect data from the patients after their recovery.

## 5. Conclusions

Examining the psychological impact of acute illnesses, such as COVID-19, on the affected individuals is important to effectively manage these conditions. Disease-associated stigma has a negative toll on individuals and societies, and measuring it using validated tools, such as the EMIC, should help in improving patient outcomes. The Arabic version of the EMIC scale has demonstrated good validity and reliability in assessing COVID-19-related stigma based on the findings of this study. However, future studies with larger sample sizes and more diverse patient populations are necessary to confirm its validity. The Arabic version of the EMIC should help researchers in the Arab world to assess disease-associated stigma among their communities and to propose policies to address this negative behavior and perception. Psychology and public health researchers in the Middle East are advised to continue exploring the validity and reliability of the EMIC scale among different patient populations from various social and economic segments as these types of studies are rare in our region. This should help define and build public health awareness campaigns to address disease-associated stigma in this part of the world.

## Figures and Tables

**Table 1:** Participants’ baseline characteristics (N = 174).

Characteristic	N (%)
**Gender**	
Female	95 (54.6)
Male	79(45.4)
**Age in years**	
18–≤25	64 (36.8)
>25–≤35	55 (31.6)
>35–≤45	35 (20.1)
>45–≤55	15 (8.6)
>55–≤65	5 (2.9)
**Marital status**	
Single	75 (43.1)
Married	92 (52.9)
Divorced	3 (1.7)
Widowed	4 (2.3)
**Nationality (Non-Saudi)**	22 (12.6)
**Number of family members**	
1–4	65 (37.4)
5–6	47 (27.0)
7–8	42 (24.1)
>8	20 (11.5)
**Employment status (Unemployed)**	35 (20.2)
**Monthly income in United States Dollars (USD)**	
≤800	73 (42.7)
800–1333.3	23 (13.5)
1333.3–1866.6	11 (6.4)
1866.6–2666.6	24 (14.0)
2666.6–4000.0	21 (12.3)
4000.0–5333.3	14 (8.2)
5333.3–8000	4 (2.3)
>8000	1 (0.6)
Refrained from answering	3 (1.7)
**Region of residence**	
Central	92 (52.9)
Western	23 (13.2)
Eastern	41 (23.6)
Southern	16 (9.2)
Northern	2 (1.1)
**Education**	
Nonformal education	3 (1.7)
Elementary	4 (2.3)
Intermediate	11 (6.3)
Secondary	36 (20.7)
Associate degree	22 (12.6)
Bachelor degree	94 (54.0)
Postgraduate degree (e.g., master of science or doctorate in philosophy)	4 (2.4)
**Participants with limited health literacy**	27 (15.5)
**Chronic health conditions**	
Diabetes	9 (5.2)
Dyslipidemia	4 (2.3)
Cardiovascular disease	7 (4.0)
Asthma	7 (4.0)
Hypothyroidism	3 (1.7)
**Participant with family history of mental illness (e.g., depression/anxiety)**	30 (17.2)

Data presented as frequency (%).

**Table 2:** Mean scores of EMIC items.

EMIC Item	Mean ± SD
EMIC-1	0.75 ± 1.18
EMIC-2	0.43 ± 1.03
EMIC-3	0.24 ± 0.72
EMIC-4	0.44 ± 0.96
EMIC-5	0.25 ± 0.71
EMIC-6	0.84 ± 1.15
EMIC-7	1.24 ± 1.28
EMIC-8	0.86 ± 1.19
EMIC-9	0.26 ± 0.68
EMIC-10	0.41 ± 0.94
EMIC-11a	0.09 ± 0.43
EMIC-11b	0.12 ± 0.44
EMIC-12	0.08 ± 0.43
Overall score	5.91 ± 5.21

**Table 3:** Extracted factors from the Arabic version of the EMIC.

Items	Factors				Communality Estimates (h2)
	Factor-1	Factor-2	Factor-3	Factor-4	
EMIC-1	0.67				0.88
EMIC-2				0.74	0.84
EMIC-3		0.82			0.71
EMIC-4		0.70			0.86
EMIC-5	0.65				0.64
EMIC-6				0.90	0.87
EMIC-7				0.72	0.88
EMIC-8			0.94		0.93
EMIC-9		0.77			0.67
EMIC-10			0.79		0.77
EMIC-11a	0.98				0.97
EMIC-11b	0.67				0.88
EMIC-12	0.98				0.97

**Table 4:** Multiple linear regression for the association of EMIC stigma score and different factors.

Variables	*β*-Estimates	95% Confidence Interval (CI)		*p*-Value *
		Lower CI	Upper CI	
Age	−0.11	−0.21	−0.01	0.03
Gender (female)	−0.90	−2.67	0.87	0.31
Health literacy	−1.80	−3.96	0.36	0.10
Education	−0.04	−0.75	0.67	0.91
Monthly income	−0.18	−0.72	0.36	0.50
Employment status (Unemployed)	−1.24	−3.63	1.15	0.31
Presence of comorbidities	−0.33	−2.45	1.80	0.76
Family history of mental illness	2.38	0.29	4.46	**0.025**
Marital status (married)	2.93	0.88	4.98	**0.005**

* Values in bold represent significant *p*-values at <0.05.

## Data Availability

The data are available upon reasonable request from the corresponding author.

## Appendix A

**Arabic Translated Explanatory Model Interview Catalogue (EMIC) Stigma Scale**

**(مقياس وصمة العار باستخدام كتالوج المقابلات التوضيحية النموذجية)**

1. **If possible, would you prefer to keep people from knowing about your COVID-19 infection? (هل تُفضل عدم معرفة الناس بإصابتك بفايروس كورونا المستجد؟)**
  - Yes. (نعم)
  - Possible. (ربما)
  - Uncertain. (لست متأكد)
  - No. (لا)
2. **Have you discussed your COVID-19 infection with the person you consider closest to you, the one whom you usually feel you can talk to most easily? (هل ناقشت إصابتك بهذا الفايروس مع الشخص الذي تعتبره أقرب إليك؛ يعني الشخص الذي تشعر أنه يمكنك ان تتحدث إليه بسهولة؟)**
  - Yes. (نعم)
  - Possible. (ربما)
  - Uncertain. (لست متأكد)
  - No. (لا)
3. **Do you think less of yourself because of your COVID-19 infection? Has it reduced your pride or self-respect? (هل شعرت بالدونية بسبب إصابتك بكورونا؟ هل قلل من كبريائك واحترامك لذاتك؟)**
  - Yes. (نعم)
  - Possible. (ربما)
  - Uncertain. (لست متأكد)
  - No. (لا)
4. **Have you ever been made to feel ashamed or embarrassed because of your COVID-19 infection? (هل أحسست بالخجل أو الإحراج بسبب إصابتك بالكورونا ؟)**
  - Yes. (نعم)
  - Possible. (ربما)
  - Uncertain. (لست متأكد)
  - No. (لا)
5. **Do your neighbors, colleagues or others in your community have less respect for you because of your infection? (هل قل احترام جيرانك وزملائك أو غيرهم في مجتمعك لك بسبب إصابتك بالكورونا؟)**
  - Yes. (نعم)
  - Possible. (ربما)
  - Uncertain. (لست متأكد)
  - No. (لا)
6. **Do you think that contact with you might have any bad effects on others around you even after you have been recovered? (هل شعرت بأن الاحتكاك بك قد يكون له أي آثار سلبية على الآخرين من حولك حتى بعد شفائك؟)**
  - Yes. (نعم)
  - Possible. (ربما)
  - Uncertain. (لست متأكد)
  - No. (لا)
7. **Do you feel others have avoided you because of your COVID-19 infection? (هل شعرت بأن الآخرين تجنبوك بسبب إصابتك بالكورونا ؟)**
  - Yes. (نعم)
  - Possible. (ربما)
  - Uncertain. (لست متأكد)
  - No. (لا)
8. **Would some people refuse to visit your home because of your COVID-19 infection even after you have been recovered? (هل رفض بعض الناس زيارة بيتك بسبب بسبب إصابتك بالكورونا حتى بعد شفائك؟)**
  - Yes. (نعم)
  - Possible. (ربما)
  - Uncertain. (لست متأكد)
  - No. (لا)
9. **If they knew about it would your neighbors, colleagues or others in your community think less of your family because of your COVID-19 infection? (لو كان الناس من حولك على علم بإصابتك بفيروس كورونا المستجد، فهل تشعر أنه قد يقلل من قدر وصورة عائلتك أمامهم؟)**
  - Yes. (نعم)
  - Possible. (ربما)
  - Uncertain. (لست متأكد)
  - No. (لا)
10. **Do you feel that your COVID-19 infection might cause social problems for your children or household in the community? (هل تشعر أن إصابتك بالكورونا قد تؤثر سلبًا على حياة أطفالك أو أفراد عائلتك في المنزل الاجتماعية؟)**
  - Yes. (نعم)
  - Possible. (ربما)
  - Uncertain. (لست متأكد)
  - No. (لا)
11. **Do you feel that this disease has caused, or will cause, problems for you to get married? (If you are married, skip this question) (هل تشعر أن هذا الفايروس قد يقلل من فرصك للزواج؟ (هذا السؤال للعزاب فقط)))**
  - Yes. (نعم)
  - Possible. (ربما)
  - Uncertain. (لست متأكد)
  - No. (لا)
12. **Do you feel that this disease has caused problems in your marriage? (If you are unmarried, please skip this question) (هل شعرت أن إصابتك بالكورونا سببت لك مشاكل في زواجك؟ (للمتزوجين فقط)))**
  - Yes. (نعم)
  - Possible. (ربما)
  - Uncertain. (لست متأكد)
  - No. (لا)
13. **Do you feel that this disease makes it difficult for someone else in your family to marry? (هل تشعر أن إصابتك بالكورونا يقلل فرص الزواج لأفراد عائلتك؟)**
  - Yes. (نعم)
  - Possible. (ربما)
  - Uncertain. (لست متأكد)
  - No. (لا)
